# *CLCN2*-related leukoencephalopathy: a case report and review of the literature

**DOI:** 10.1186/s12883-019-1390-7

**Published:** 2019-07-10

**Authors:** Zhuoxin Guo, Tingting Lu, Lisheng Peng, Huanhuan Cheng, Fuhua Peng, Jin Li, Zhengqi Lu, Shaoqiong Chen, Wei Qiu

**Affiliations:** 1 Department of Radiology, The Third Affiliated Hospital of Sun Yat-sen University, No. 600 Tianhe Road, Tianhe District, Guangzhou, China; 2 Department of Neurology, The Third Affiliated Hospital of Sun Yat-sen University, No. 600 Tianhe Road, Tianhe District, Guangzhou, China; 3 Department of Ophthalmology, The Third Affiliated Hospital of Sun Yat-sen University, No. 600 Tianhe Road, Tianhe District, Guangzhou, China

**Keywords:** ClC-2 chloride channels, Leukoencephalopathies, MRI, Diffusion tensor imaging

## Abstract

**Background:**

Loss-of-function mutations in the *CLCN2* gene were recently discovered to be a cause of a type of leukodystrophy named *CLCN2*-related leukoencephalopathy (CC2L), which is characterized by intramyelinic edema. Herein, we report a novel mutation in *CLCN2* in an individual with leukodystrophy.

**Case presentation:**

A 38-year-old woman presented with mild hand tremor, scanning speech, nystagmus, cerebellar ataxia in the upper limbs, memory decline, tinnitus, and dizziness. An ophthalmologic examination indicated macular atrophy, pigment epithelium atrophy and choroidal capillary atrophy. Brain magnetic resonance imaging (MRI) showed the diffuse white matter involvement of specific white matter tracts. Decreased diffusion anisotropy was detected in various brain regions of the patient. Diffusion tensor tractography (DTT) showed obviously thinner tracts of interest than in the controls, with a decreased fiber number (FN), increased radial diffusivity (RD) and unchanged axial diffusivity (AD). A novel homozygous c.2257C > T (p.Arg753Ter) mutation in exon 20 of the *CLCN2* gene was identified.

**Conclusion:**

CC2L is a rare condition characterized by diffuse edema involving specific fiber tracts that pass through the brainstem. The distinct MRI patterns could be a strong indication for CLCN2 gene analysis. The findings of our study may facilitate the understanding of the pathophysiology and clinical symptoms of this disease.

**Electronic supplementary material:**

The online version of this article (10.1186/s12883-019-1390-7) contains supplementary material, which is available to authorized users.

## Background

*CLCN2*-related leukoencephalopathy (CC2L), also known as leukoencephalopathy with ataxia (LKPAT; MIM #615651), is a rare autosomal recessive disorder caused by mutations in the *CLCN2* gene (600570) on chromosome 3q27.1, causing the dysfunction of its encoded ClC-2 chloride channel protein, which is characterized by intramyelinic edema [1–3]. The molecular background of CC2L remains elusive. Since the identification of CC2L in 2013 by Depienne et al., only 13 cases have been reported in detail. The majority of patients show mild clinical phenotypes with prolonged survival. Herein, we report a novel and pathogenic variant of *CLCN2* in a woman with leukodystrophy and visual impairment. For the first time diffusion tensor imaging (DTI) technique was employed to explore the microstructural changes of this disease.

## Case presentation

**Fig. 1:**
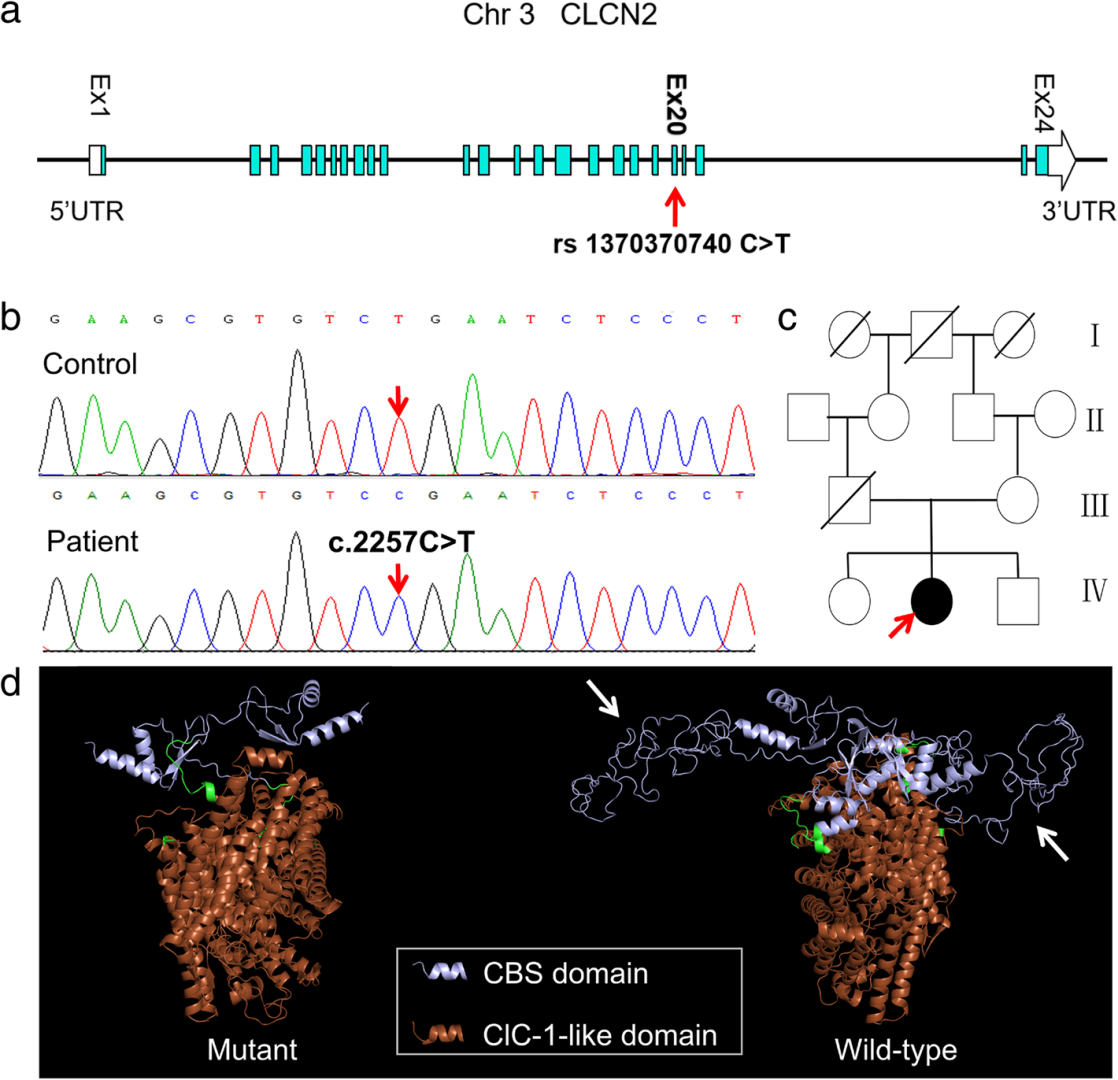
Pedigree and molecular findings of the patient. **a** Schematic representation of *CLCN2* on chromosome 3q27.1 showing a novel homozygous nonsense mutation located in exon 20. **b** Sequencing chromatograms of this mutation. **c** Pedigree of the patient. Her father died of brain glioma. **d** Ribbon diagrams of the predicted CLCN2 structure from wild-type and p.Arg753Ter mutant protein. Some structures (arrows) in the cystathionine beta-synthase (CBS) domain of the mutant protein are missing

A 38-year-old woman presented with mild intention tremor of her hands that had developed at the age of 22. This symptom had been slowly progressive and had been accompanied with speech impediments characterized by a slow rate of speech and a flat voice since the age of 37. The patient complained of mild memory decline, mild tinnitus in both ears, and occasional dizziness. From a young age, she had experienced poor vision in both eyes, which had gradually worsened. She had a history of hyperthyroidism and had been disease free before the onset of neurologic symptoms. The patient was born of a consanguineous union. The family diagram is presented in Fig. 1c.

A neurological examination indicated scanning speech, horizontal nystagmus in both eyes, cerebellar ataxia and postural tremor in the upper limbs at a frequency of approximately 8 Hz. Bilateral prolonged latency and a slightly reduced amplitude of the P100 wave in the visual evoked potential and central injury in the brainstem auditory evoked potentials were detected. The visual acuity was 0.15 in the right eye and 0.10 in the left eye, which was not corrected by eyeglasses. Optical coherence tomography (OCT) indicated macular atrophy, especially in the outer segment layer. Fundus fluorescence angiography (FFA) showed strong macular fluorescence changes, indicating pigment epithelium atrophy, and spots inside that were lacking fluorescence, indicating choroidal capillary atrophy. Thyroid-stimulating hormone and parathyroid hormone levels were slightly elevated. Examinations of cognitive function and motor and somatosensory evoked potentials were normal.

**Fig. 2:**
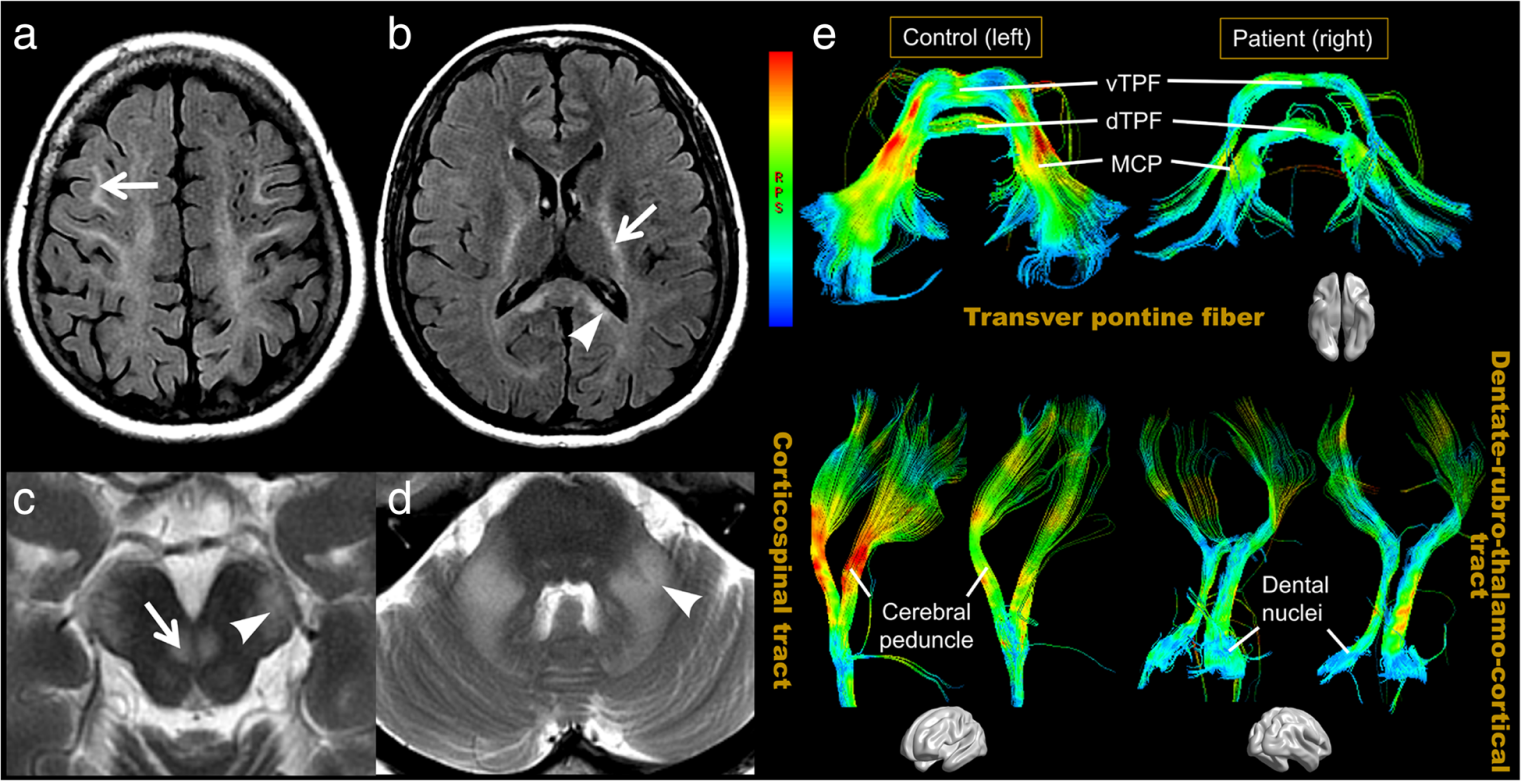
Conventional brain magnetic resonance imaging (MRI) and diffusion tensor tractographies (DTTs) of the patient. Axial T2-FLAIR (**a**, **b**), and T2-weighted (**c**, **d**) MRI of the patient demonstrate abnormal hyperintensities in the bilateral frontal (**a**, arrow) and parietal white matter and the splenium (arrowhead, **b**) of the corpus callosum, internal capsule (arrow, **b**), cerebral peduncle (arrowhead, **c**), DSCP (arrow, **c**), and middle cerebellar peduncle (arrowhead, **d**). On DTT (**e**), the CST, transverse pontine fibers, and dentate nucleus tracts of the patient are obviously thinner and have a “darker color” in the pathological tracts, which indicates lower fractional anisotropy (FA) values, when compared with those in a control. *DSCP = decussation of the superior cerebellar peduncle, CST = corticospinal tract, vTPF = ventral transverse pontine fiber, dTPF = dorsal transverse pontine fiber, MCP = middle cerebellar peduncle*

Conventional magnetic resonance imaging (MRI) showed confluent white matter abnormalities with hypointense T1-weighted and hyperintense T2-weighted signals, with the symmetrical involvement of the internal capsules, cerebral peduncles, and middle cerebellar peduncles (Fig. 2a-d). Diffusion-weighted imaging (DWI) showed hyperintensity in the pathological areas, with no restrictions on the apparent diffusion coefficient (ADC) map. No enhanced lesion was found on the post-gadolinium T1-WI sequence. The brain atrophy was unremarkable. Brain DTI images were acquired and compared to those from three gender- and age- (less than 5 years apart) matched healthy controls. Decreased fractional anisotropy (FA) values were found in almost all regions with white matter hyperintensity (WMHI), as well as in specific structures of normal-appearing white and gray matter [see Additional file 1]. A reduced fiber number (FN) was detected in areas with obvious FA reductions, when compared with that in the controls [see Additional file 1]. The findings also revealed decreased axial diffusivity (AD) in the optic nerves, and increased radial diffusivity (RD) in the middle cerebellar peduncles and cerebral peduncles of the patient. On diffusion tensor tractography (DTT), the white matter tracts of interest were visibly thinner than those in the controls, as illustrated in Fig. 2e.

Whole-exome sequencing revealed a novel homozygous c.2257C > T (p.Arg753Ter) mutation in exon 20 of the *CLCN2* gene, which was a nonsense mutation that altered the 753rd Arg in the encoding protein and generated a stop codon (Fig. 1a, b). This change was not reported in any genetic database. Homology modelling suggested the disruption of cystathionine beta-synthase (CBS) domain by this variant (Fig. 1d), which was considered to be pathogenic. The patient’s mother was a heterozygous carrier of this mutation.

## Discussion and conclusions

### ClC-2: a brief overview

ClC-2 is a type of permeable channel for chloride ions that spans the cell membrane [4, 5]. This protein shows widespread tissue expression, including in the brain, pancreas, kidney, liver, hearts, lungs, skeletal muscles and gastrointestinal tract [4–6]. In the central nervous system, ClC-2 mainly localizes to the endfeet of GFAP-positive astrocytes that surround the blood vessels, to the gap junctions along the circumference of oligodendrocytes, and to pyramidal neurons in the hippocampus [1, 3, 7]. Under physiological conditions, ClC-2 plays an important role in transepithelial transport, ion homeostasis, and the regulation of cell excitability [4, 6]. The disruption or abnormality of ClC-2 may affect these organs or tissues, causing various disease conditions, namely, channelopathies [6]. Humans harboring loss-of-function *CLCN2* variants and *Clcn2* knockedout (KO) mice share overlapping phenotypes, mainly including leukoencephalopathy, male infertility, and visual impairment. However, gain-of-function *CLCN2* mutations have been suggested as a cause of a substantial fraction of familial hyperaldosteronism type II [7].

### Research on the molecular basis of CC2L

The molecular background of CC2L remains elusive. Functional and biochemical analyses indicated that *Clcn2* mutations could cause defective ClC-2 functional expression, probably through a complex mechanism involving reduced cellular and plasma membrane density, increased turnover, and impaired gating of ClC-2 [8, 9]. A reduction in the ClC-2-mediated currents was observed in oligodendrocytes of *Clcn2* KO mice and in A500V-ClC-2 expressing mammalian cells [8, 10], supporting the ideas of Depienne et al., who suggested that ClC-2 disruption might result in the disturbance of the compensation of action-potential-induced ion shifts [1], leading to osmotic intramyelinic edema [3]. Recent studies have shown that GlialCAM/MLC1 complex, which physically binds ClC-2 [11, 12], increased the activity of A500V-ClC-2 [8], while the additional disruption of GlialCAM aggravated the vacuolization in *Clcn2* KO mice [10]. Together, these findings indicate that functions of the mutant ClC-2 protein are at least partly regulated by the GlialCAM/MLC1 by unknown molecular mechanisms possibly involved in the pathophysiology of CC2L.

### Clinical features

The clinical manifestations of the reported patients and the *CLCN2* variants that they carried are summarized in Table 1 and Table 2, respectively. Our patient displayed a clinical phenotype similar to the previous cases. However, she also developed asymptomatic hyperthyroidism and hyperparathyroidism with unknown causes, which were not previously seen in those cases. In consideration of the high expression of ClC-2 in the glands [4], the relationship between *CLCN2* mutations and the abnormal function of the thyroid and parathyroid should be investigated in future studies.

**Table 1:** Clinical features of the reported patients with *CLCN2*-related leukoencephalopathy

Features	No. of subject
Gender (%)	
Female	12 (86)
Male	2 (14)
Age at first sign	3 months - 57 years (27.87 ± 18.82)
Presenting signs (%)	
Ataxia	5 (36)
Headache	4 (29)
Action tremor	3 (21)
Visual changes	3 (21)
Psycho-cognitive problems	2 (14)
Tinnitus and vertigo	1 (7)
Limbs numbness	1 (7)
Azoospermia	1 (7)
Seizure	1 (7)
Motor dysfunction (%)	13 (93)
Cerebella ataxia	11
Action tremor	5
Spasticity	2
Seizure	1
Paraparesis	1
Psycho-cognitive problems (%)	5 (36)
Learning disability	3
Psychosis	1
Mild memory decline	1
Mild executive dysfunction	1
Headache (%)	5 (36)
Ocular changes (%)	4 (29)
Uncorrectable diminished visual acuity	3
Optic neuropathy	3
Retinopathy	4
Visual field defects	4
Abnormal evoked potential (%)	3 (21)
Brainstem auditory evoked potential	2
Visual evoked potential	2
Motor evoked potential	1
Auditory abnormality (%)	3 (21)
Hearing loss	2
Tinnitus	2
Male infertility (%)	1 (50)
Consanguineous parents (%)	7 (50)

**Table 2:** *CLCN2* mutations of the reported patients with *CLCN2*-related leukoencephalopathy

Case	Exon	DNA	Protein	Genotype
1	1	c.61dup	p.Leu21ProfsTer27	Homozygous
2	–	c.1412G > A	p.Arg471His	Homozygous
3	–	–	p.Glu475LysfsTer79	Homozygous
4	–	–	p.Leu435ArgfsTer7	Compound heterozygous
5	16	c.1769A > C	p.His590Pro	Homozygous
6	–	c.1113delinsACTGCTCAT	p.Ser375CysfsX6	Homozygous
7	–	c.1507G > A	p.Gly503Arg	Homozygous
8	15	c.709G > A	p.Trp570X	Homozygous
9	15	c.1709G > A	p.Trp570X	Homozygous
10	4	c.430_435del	p.Leu144_Ile145del	Homozygous
11	11; 2 to part of 6	c.1143delT; c.64–1107_639del	p.Gly382AlafsX34; p.Met22LeufsX5	Heterozygous; heterozygous
12	14	c.1499C > T	p.Ala500Val	Homozygous
13	8	c.828dupG	p.Arg277AlafsX23	Homozygous
14	20	c.2257C > T	p.Arg753Ter	Homozygous

1. Hoshi et al. [2018]

2–4. Zeydan et al. [2017]

5. Giorgio et al. [2017]

6. Hanagasi et al. [2015]

7. Di Bella et al. [2014]

8–13. Depienne et al. [2013]

14. The case of our study

Cerebellar ataxia is a predominant finding and is the most common initial symptom of this disease. The presentation of cerebellar ataxia presents varied, including gait ataxia, intention tremor (commonly in the hands), nystagmus, dysmetria and dysarthria. The neurologic deficits are mild, lack specific manifestations, and are stable or have a slowly progressive course, possibly leading to a delay in diagnosis. However, as specific clinical patterns of this disease, retinopathy and male infertility may be diagnostic clues that prompt, to a certain extent, a clinical suspicion of CC2L. The diagnosis relies on confirmatory genetic testing. Generally, CC2L has a favourable prognosis, and no disease-related blindness, permanent mobility impairment or deaths have been reported to our knowledge. To date, there is no specific treatment for CC2L, and only supportive care is available [2].

### Conventional MRI techniques

The brain abnormalities showed confluent, symmetric T_2_-weighted hyperintensities along the fiber tracts, without contrast enhancement. The major criteria are the involvement of the posterior limbs of the internal capsules, cerebral peduncles, and middle cerebellar peduncles. In addition, the supportive criteria include involvement of the following: the pyramidal tracts in the pons, the central tegmental tracts in the brainstem, the superior cerebellar peduncles, decussation of the superior cerebellar peduncles, corpus callosum, dentate nucleus, and other white matter of the cerebrum and cerebella [2]. The periventricular white matter is relatively unaffected. Two subclinical patients with CC2L with incidentally found leukoencephalopathies have been reported, including a 42-year-old Italian man with the onset of azoospermia [13], and a 52-year-old Moroccan woman presenting with optic atrophy [9]. This finding suggested that the brain MRI abnormalities of CC2L might precede the neurologic symptoms. CC2L has a quite distinct MRI phenotype that may facilitate early genetic testing and diagnosis. This disease can be identified from the inherited vasculopathies with white matter involvement, inflammatory demyelinating diseases or certain leukodystrophies that show multifocal white matter changes or contrast-enhancing lesions.

### Advanced MRI techniques

DWI shows hyperintensity in the pathological areas, with restricted diffusion in the ADC maps of some patients [1, 14–16] and with no restricted diffusion in others [9, 13], including in the patient in the current study. This discrepancy may be explained in part by the differences in the free water content, which could be influenced by the size of the myelin vacuoles and extracellular space in the lesions [1, 13]. In the ClC-2 KO brain, the vacuole size slowly increased with the age of the mice [3]. The longitudinal data describing the radiological course of CC2L are limited. In a 42-year-old man with a homozygous missense *CLCN2* mutation, the leukodystrophic pattern was unchanged over a 2-year follow-up [13]. However, in a 22-month-old female with a homozygous frameshift *CLCN2* mutation, abnormal signals had spread at a 4-month follow-up DWI, and some of those abnormalities had disappeared at a 10-month follow-up [16].

Decreased diffusion anisotropy was detected in various brain regions on the FA map of the patient, which was more widespread than the WMHI on the conventional MRI. Interestingly, some of the abnormal regions highly overlapped with the constituents of the inputs and outputs of the red nucleus [17–19], namely, dentate-rubro-thalamo-cortical tracts (DRT) and corticorubrospinal tracts (CRST) respectively. Patients suffering from lesions involving the aforementioned tracts could present with ataxia and tremor [20–22]. Therefore we speculated that there may be a link between the cerebellar ataxia of the patient and the involvement of red nucleus connectivity. This hypothesis needs to be confirmed with further studies based on a larger number of cases. Additionally, our results suggested that the FA map was able to demonstrate the subtle changes of CC2L that may be missed with conventional MRI. This could be a valuable tool for the early diagnosis of subclinical patients.

DTT showed that the cerebral peduncle and middle cerebellar peduncle of the patient were obviously thinner than those of the controls, and the FN values decreased. This observation raises the question of whether axonal degeneration or damage exists in pathological tracts. However, further investigation of the directional diffusivities of these structures revealed increased RD and unchanged AD. Increased RD and decreased AD are markers of myelin and axonal injury, respectively [23–25]. Therefore, our results suggest that there was damaged myelin integrity and unaffected axons, supporting the observations in ClC-2 KO mouse [3, 26]. A possible explanation for the thinned pathological tracts on DTT is that the markedly affected tracts were incapable of being tracked under the present processing threshold (FA > 0.20, fiber angulation < 70°) due the pressure of the prominent vacuoles. The relative preservation of axons could partly explain the mild neurologic symptoms of the patient despite significant white matter involvement. However, we could not rule out the possibility of accompanying mild axonal damage. Human neuropathology should be performed to verify our findings.

In contrast, decreased AD and FN but unchanged RD were observed in the optic nerves of the patient, suggesting optic atrophy and the preservation of myelin. In addition, pigment epithelium atrophy and choroidal capillary atrophy was observed on FFA of the patient, as well as macular atrophy on OCT. These results are in line with those of previous studies, which suggest that *Clcn2* mutations could lead to pigment epithelium cells dysfunction and cause a severe early-onset retinal degeneration, and no vacuole was observed in the optic nerves [3, 26, 27]. The vacuolation of the optic nerve of Cx47/32 double-KO mice could be suppressed by inhibiting optic nerve activity [3]. Therefore, it was suggested that the absence of vacuolation in the optic nerves of ClC-2 KO mice was the result of the severe retinal degeneration and the simultaneous reduction of optic nervous electric activity [3]. Furthermore, the macular atrophy and choroidal capillary atrophy of the patient, which have not been previously reported, may be kinds of disuse atrophy secondary to the retinal degeneration.

We are aware of some limitations of this study. DTI analysis was performed in only one CC2L case and on a deficient number of controls, which limits the strength of the study. The region of interest (ROI)-based method is commonly used in case studies, but it is hypothesis-driven and cannot be used for whole-brain analysis.

In conclusion, CC2L is a rare condition characterized by diffuse edema involving specific fiber tracts passing through the brainstem. The distinct MRI patterns could be a strong indication for *CLCN2* gene analysis. The findings of our study may facilitate the understanding of the pathophysiology and clinical symptoms of the disease.

## Additional file

Additional file 1: **Table S1.** describe results of fractional anisotropy measurement, and Table S2 describe results of fiber number, axial diffusivity, and radial diffusivity measurement. (docx 21 kb) (DOCX 20 kb)

## Data Availability

All data generated or analysed during this study are included in this published article [and its supplementary information files]. The funding body did not participate in the study design, data collection, analysis and interpretation, decision to publish, or preparation of the manuscript.

## Abbreviations

AD: Axial diffusivity
ADC: Apparent diffusion coefficient
CBS: Cystathionine beta-synthase
CC2L: *CLCN2*-related leukoencephalopathy
CRST: Corticorubrospinal tracts
CST: Corticospinal tract
DRT: Dentate-rubro-thalamo-cortical tracts
DSCP: Decussation of superior cerebellar peduncle
DTI: Diffusion tensor imaging
dTPF: Dorsal transverse pontine fiber
DTT: Diffusion tensor tractography
DWI: Diffusion weighted imaging
FA: Fractional anisotropy
FN: Fiber number
KO: Knocked out
LKPAT: Leukoencephalopathy with ataxia
MCP: Middle cerebellar peduncle
MRI: Magnetic resonance imaging
RD: Radial diffusivity
ROI: Region of interest
vTPF: Ventral transverse pontine fiber
WMHI: White matter hyperintensity
