# Supplementary material for: CLCN2-related leukoencephalopathy: a case report and review of the literature
Source: BMC Neurol. 2019 Jul 10;19:156. doi: 10.1186/s12883-019-1390-7 (PMC6617604; doi:10.1186/s12883-019-1390-7)
Supplement: Supplementary file 1 — Table S1. describe results of fractional anisotropy measurement, and Table S2 describe results of fiber number, axial diffusivity, and radial diffusivity measurement. (docx 21 kb) (DOCX 20 kb) [file 12883_2019_1390_MOESM1_ESM.docx]

| Region of interest (ROI) | FA | |  |
| --- | --- | --- | --- |
|  | Patient | Controls |  |
| ***White matter hyperintensity*** |  |  |  |
| Cerebral peduncle | 0.492* | 0.733 (0.023) |  |
| Middle cerebellar peduncle | 0.452* | 0.622 (0.039) |  |
| Splenium of corpus callosum | 0.687* | 0.841 (0.025) |  |
| Genu of corpus callosum | 0.715* | 0.835 (0.036) |  |
| Body of corpus callosum | 0.721* | 0.818 (0.027) |  |
| Decussation of superior cerebellar peduncle | 0.433* | 0.505 (0.021) |  |
| Ventral transverse pontine fiber | 0.428* | 0.564 (0.020) |  |
| Corticolspinal tract in pons | 0.550* | 0.605 (0.024) |  |
| Dorsal transverse pontine fiber | 0.494* | 0.583 (0.036) |  |
| Central tegmental tract in pons | 0.616* | 0.670 (0.024) |  |
| Anterior limb of internal capsule | 0.561* | 0.679 (0.018) |  |
| Posterior limb of internal capsule | 0.551* | 0.676 (0.013) |  |
| Superior cerebellar peduncle | 0.716 | 0.692 (0.047) |  |
| Frontal lobe | 0.519* | 0.594 (0.027) |  |
| ***Normal-appearing white matter*** |  |  |  |
| Optic tract | 0.351* | 0.456 (0.021) |  |
| Optic nerve | 0.319* | 0.545 (0.040) |  |
| Occipital lobe | 0.566 | 0.569 (0.032) |  |
| Temporal lobe | 0.428 | 0.404 (0.041) |  |
| Cerebellar hemisphere | 0.160* | 0.191 (0.010) |  |
| ***Gray matter*** |  |  |  |
| Red nucleus | 0.459* | 0.531 (0.023) |  |
| Substantia nigra | 0.427 | 0.428 (0.015) |  |
| Dental nucleus | 0.286 | 0.311 (0.012) |  |
| Thalamus | 0.237* | 0.367 (0.016) |  |
| Caudate nucleus | 0.137* | 0.166 (0.009) |  |
| Putamen | 0.134 | 0.113 (0.017) |  |
| Globus pallidus | 0.234 | 0.280 (0.037) |  |
| Hippocampus | 0.584* | 0.630 (0.020) |  |
| Cingulum | 0.565* | 0.656 (0.038) |  |
| * indicates abnormal decreased FA values.  Values represent as mean for the patient, and mean (SD) for the controls. | | |  |

**Table 1． Fractional anisotropy (FA) values of the patient and the controls**

Decreased FA values are found in almost all regions of white matter hyperintensity (WMHI), as well as in specific structures of the normal-appearing white matter (NAWM) and gray matter, including the optic tracts, optic nerve, cerebellar hemisphere, red nucleus, thalamus, caudate nucleus, hippocampus, and the cingulum.

FA values were determined by using the region of interest (ROI) method in the FuncTool image analysis software (GE Healthcare). The ROIs are oval-shaped, with standardized sizes for the same structure in all the subjects, and are placed bilaterally except for the decussation of the superior cerebellar peduncles and transverse pontine fiber. Comparisons were made between the patient and three gender- and age-matched healthy controls. FA within the assessed structures of the patient were defined as abnormal when the mean values are more than 2 standard deviations (SDs) lower or higher than those of the normal controls.

DTI images were acquired using spin-echo echo-planar imaging (TR/TE/thickness/FOV/matrix/NEX = 4,135ms/83ms/4mm/24cm/128×128/2) with 25 isotropically distributed orientations for diffusion-sensitizing gradients at a b-value of 1,000 ms/mm2 and 3 b=0 images.

**Table 2. Fiber number (FN), axial diffusivity (AD), and radial diffusivity (RD) measured in the regions of interest of the patient and controls**

| Region of interest  (ROI) | FN | |  | AD | |  | RD | |
| --- | --- | --- | --- | --- | --- | --- | --- | --- |
|  | patient | controls |  | patient | controls |  | patient | controls |
| Middle cerebellar  peduncle | 256 ↓ | 340  (37.9) |  | 1.312e^-004^ | 1.233e^-004^ (0.115) |  | 6.203e^-004^↑ | 4.650e^-004^  (0.244) |
| Posterior limb of  internal capsule | 295 ↓ | 451  (31.5) |  | 1.220e^-004^ | 1.217e^-004^ (0.053) |  | 5.362e^-004^ | 5.076e^-004^ (0.232) |
| Cerebral peduncle | 281 ↓ | 319  (24.6) |  | 1.258e^-004^ | 1.334e^-004^ (0.080) |  | 5.447e^-004^ ↑ | 4.702e^-004^ (0.235) |
| Optic nerve | 46 ↓ | 62  (14.0) |  | 1.579e^-004^ ↓ | 1.870e^-004^ (0.115) |  | 8.471e^-004^ | 8.895e^-004^ (1.143) |

The findings reveal decreased AD in the optic nerves, increased RD in the middle cerebellar peduncles and cerebral peduncles, and reduced FN in all the assessed structures of the patient, when compared with those in the controls.

FN, AD and RD values were obtained by using the ROI method similar to the FA measurement using DTI-Studio software (CMRM, Johns Hopkins Medical Institute, Baltimore, USA). The parameters assessed in the patient were defined as abnormal when the mean values were more than 2 standard deviations (SDs) lower or higher than those of the normal controls.
